# Analysis of the developmental stages, kinetics, and phenotypes exhibited by myeloid cells driven by GM-CSF *in vitro*

**DOI:** 10.1371/journal.pone.0181985

**Published:** 2017-07-27

**Authors:** Peter B. Rogers, Michelle G. Driessnack, Elizabeth Hiltbold Schwartz

**Affiliations:** Department of Biological Sciences, Auburn University, Auburn AL, United States of America; Oklahoma Medical Research Foundation, UNITED STATES

## Abstract

The developmental progression of conventional DC has been quite well defined, yet the developmental pathway of monocyte-derived, GM-CSF-driven DC is less well understood. We addressed this issue by establishing an isolation strategy that identifies five distinct GM-CSF derived cell types. Expression of Ly6C and CD115 (Csf-1R) was used to identify and isolate four populations. One of the populations could be further separated based on CD11c expression, distinguishing five populations. We further defined these cells based on expression of transcription factors and markers of early and later stages of myeloid development. These discreet developmental stages corresponded well with previously defined populations: Common Myeloid Progenitors (CMP), Granulocyte/Macrophage Progenitors (GMP), Monocytes, as well as Monocyte-derived macrophages (moMac) and Monocyte-derived DC (moDC). Finally, within the moMac population we also identified moDC precursor activity (moDP) that could be distinguished from moMac and moDC based on their level of MHC class II expression and developmental plasticity.

## Introduction

Dendritic cells (DC) are central to the establishment of adaptive immune responses and offer great promise as vehicles for vaccination and therapies for a variety of diseases [1–4]. Culture of cytokine differentiated DC from mouse bone marrow has also enabled the study of the molecular mechanisms utilized by these cells for pathogen recognition, antigen processing and presentation, and T cell priming. Large numbers of DC can be generated by culturing bone marrow in cytokines such as Flt3L or GM-CSF [5–8]. DC generated from mouse bone marrow in culture with GM-CSF (GMDC) phenotypically and functionally reflect inflammatory DC in vivo elicited by a variety of infections [9–11]. The developmental progression of cells differentiated in Flt3L has been well studied [12–16], yet the development and differentiation of GM-CSF-driven, or monocyte-derived DC (moDC), is less well understood. Thus, the developmental stages at which specific phenotypes and functions are acquired during this process remain to be determined.

The developmental program of DC occurs through a set of sequential steps, at each of which, the cells express a unique profile of transcription factors and characteristic cell surface markers [13,17,18]. Several developmental progenitors and precursors of conventional DC have been identified, including MDP (monocyte-macrophage dendritic progenitor) [19–21] and CDP (common dendritic precursor) [22–25]. In the developmental pathway of GM-CSF-driven or monocyte-derived DC (moDC), the early stages of development include common myeloid progenitors (CMP) [26,27], which give rise to granulocyte macrophage progenitors (GMP) [15,28], followed by monocytes. A precursor of monocytes and macrophages but not dendritic cells (cMOP) has also been identified in the bone marrow [19], yet its place in the GM-CSF-driven differentiation pathway remains to be determined. Furthermore, while moDC are known to derive from monocytes [29], the later developmental checkpoints that have been identified in cDC, known as preDCs have not been identified for this lineage. Specifically, it is not clear if there is a correlate of the preDC in the inflammatory DC lineage between monocytes and IDC.

Thus, with this study, we set out to better define the sequential development of myeloid cells on the path to DC differentiation driven by GM-CSF in vitro. We have developed a sorting strategy based on the expression of two key phenotypic/functional markers (Ly6C and CD115). This strategy has enabled identification of five developmentally distinct cell stages, which represent CMP, GMP, Monocytes, and two more differentiated CD11c^+^MHCII^+^ cell types, moMac (a population resembling GM-Macs recently described by Helft, et al [30]) and traditional moDC. We also observed DC precursor activity in the population that shares the phenotype of moMac, and have termed this cell type moDP (Monocyte-derived DC Precursor). Adoptive transfer studies confirm that this GM-CSF driven developmental progression is also observed in vivo. This advance in our understanding of moDC development will support the use of these cells as clinical therapies providing better ways to isolate and identify specific developmental stages with ideal functional characteristics.

## Materials and methods

### Mice

This work is approved by an in full compliance with the Institutional Care and Use Committee of Auburn University regarding the use of animals. C57BL/6 and B6.SJL-Ptprc^a^ Pepc^b^/BoyJ mice were purchased from Jackson Laboratories. Mice were euthanized prior to bone marrow harvest by CO_2_ narcosis in accordance with the rules established by the 2013 American Veterinary Medical Association (AVMA) Guidelines on Euthanasia. To ensure irreversibility of the euthanasia process, cervical dislocation was performed following CO_2_ euthanization

### DC propagation

Bone marrow DC were generated as previously described [5]. Briefly, bone marrow was removed from the femurs and tibias of C57BL/6 mice. Following red cell lysis, cells were grown in RPMI 1640 medium supplemented with 10% fetal calf serum, glutamine, 2-mercaptoethanol, and 10ng/mL of recombinant granulocyte/macrophage-colony stimulating factor at a density of 1x10^6^ cell/mL. Cells were incubated at 37°C in 5% CO_2_ and fed with fresh media every two days.

### Flow cytometry

Fluorescently conjugated antibodies against mouse surface antigens were used to measure expression. Anti-Ly6C (clone HK1.4), anti-MerTK (clone DS5MMER), anti-CD34 (clone HM34), and anti-I-A^b^ (clone AF6-120.1), were obtained from eBioscience. Anti-CD115 (clone AF598), anti-Sca-1 (clone E13-161.7), Ly6G (clone HK1.4), anti CD64 (clone x54-517.1), and anti-CD11b (clone 1D4B) were obtained from Biolegend. Anti-CD117 (clone 2B8), anti-CD14 (clone RMC5-3), anti-CD16/32 (clone 2.4G2), anti-Gr-1 (clone RB6-7C5), anti-CD172a (clone 1D4B), anti-CD11c (clone HL3), anti-CD40 (clone 3/23), anti F4/80 (clone T45-2342), and anti-CD86 (clone GL1) were obtained from BD. The level of fluorescence was determined by flow cytometry using a BD Accuri^TM^ C6 flow cytometer and analyzed using FlowJo® software. Fluorescence Minus One (FMO) controls were generated by staining cells with only Ly6C and CD115 and measuring the fluorescence in the un-stained channel.

Cell sorting was performed on day 3 or day 5 based on a panel of either Ly6C, CD115, and CD11c expression, or CD11b, MHC II, and CD11c expression. All cell sorting was performed using a Cytomation MoFlo® XDP High-Speed Cell Sorter. Double sort analysis was performed by isolating Ly6C^-^CD115^+^ cells on day 3, culturing in GM-CSF supplemented media, and isolating the downstream Ly6C^-^CD115^+^ and Ly6C^-^CD115^-^ populations 6 days post initial sort. Gating strategy for exclusion of doublets and sorting is depicted in S1 Fig.

### Magnetic-associated cells sorting

Anti-APC Multisort Kit (Milteyi Biotec; #130-091-255), Ly6C-APC (eBiosciences; clone HK1.4), CD115 Microbead Kit (Milteyi Biotec; #130-096-354), and LD Columns (Milteyi Biotec; #130-042-901) were used. Murine bone marrow cells were harvested and cultured for 2 days as previous described. 3x10^7^ cells were recovered and sorted according to manufacturer’s instructions. Cells were treated with FC blocking buffer and stained with Ly6C-APC and CD115-biotin. After incubation and washing, cells were incubated with anti-APC beads and passed through an LD column. The flow through and retained factions were collected, and anti-APC beads were cleaved with Release Buffer. Both factions were then incubated with Stop Buffer and anti-Biotin beads. The two factions were passed through a second LD column, resulting in 4 fractions based on Ly6C and CD115 profiles.

### Gene expression analysis

RNA was isolated from sorted populations using RNAqueous®-4PCR kit, and qPCR was performed using a custom RT^2^ Profiler PCR array from Qiagen® according to manufacturer’s instructions. The following transcipts were analyzed: Id2, Irf8, Irf4, Stat3, Stat5b, Spi1, Nfkb1, Batf3, Gfi1, Cebpa, Ciita, Irf2, Cx3cr1, Tcf7l2, Cebpe, Pecam, Cd34, Kit, Flt3, Relb, Klf4, Zbt46, Runx2, Zfp367, Pml, Csfr3, Rn18s, and Gapdh.

### Co-Culture and adoptive transfer experiments to track developing myeloid cells in vitro and in vivo

Bone marrow was harvested from Ptprc^b^ (CD45.1) mice and cultured in GM-CSF supplemented media for 1 or 4 days. Cells were sorted based on expression of CD11c, Ly6C, and CD115. In co-culture assays, 10^4^ CD45.1^+^ sorted cells were co-cultured with 10^6^ CD45.2^+^ fresh bone marrow cells supplemented with GM-CSF. Adoptive transfers were performed by intraperitoneal injection of 10^6^ sorted CD45.1^+^ cells suspended in PBS with 200ng of GM-CSF into CD45.2^+^ mice. Mice received daily injections of 200ng of GM-CSF. Peritoneal lavage was collected every 48 hours.

## Results

### Differential kinetics of Ly6C and CD115 expression allows for identification of developmentally distinct populations of GM-CSF driven myeloid cells

**Fig 1:**
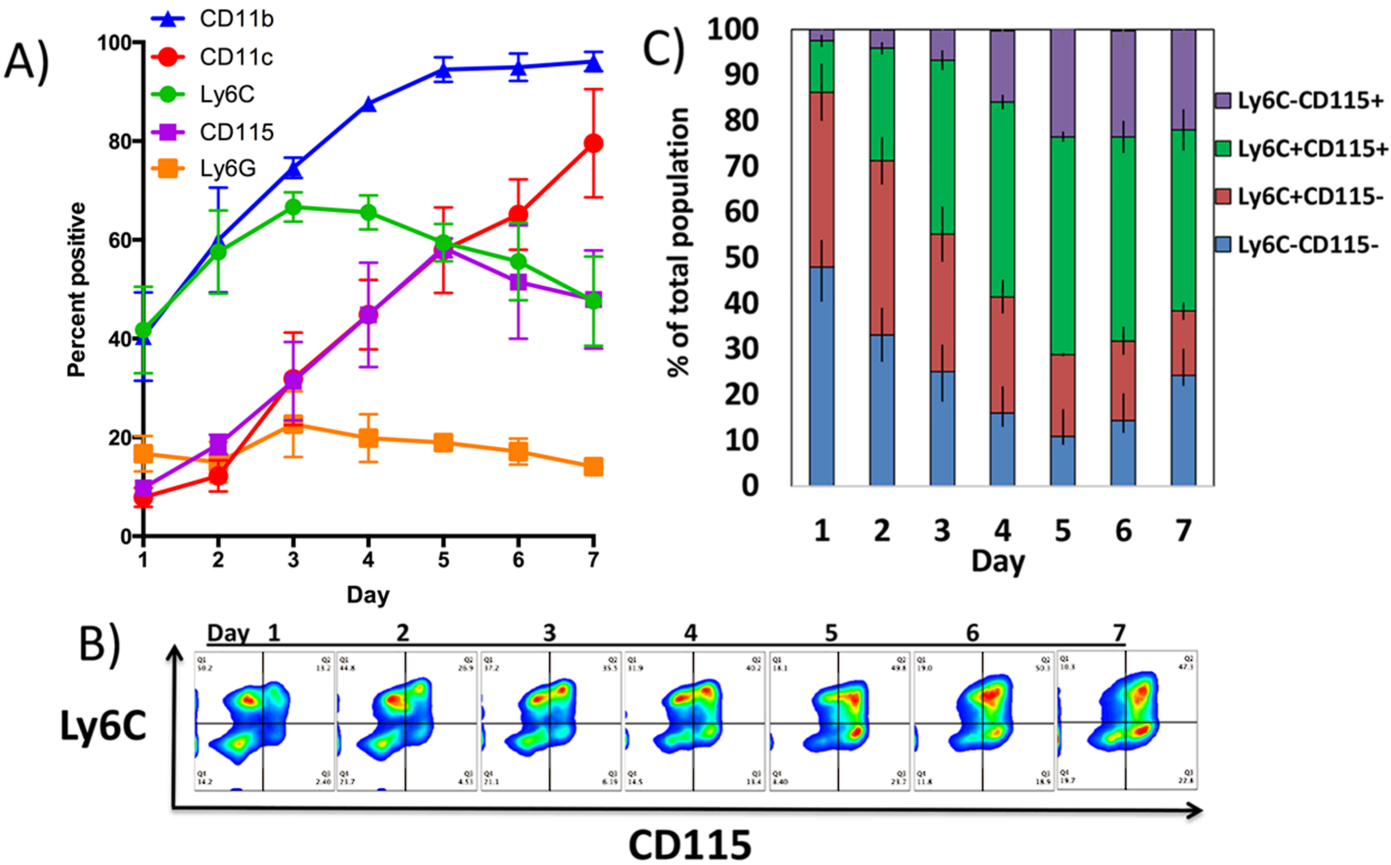
Expression of myeloid and dendritic cell markers by GM-CSF driven bone marrow cells over seven days in culture. Bone marrow cells were cultured in GM-CSF for seven days. A) Expression of CD11b, CD115, Ly6C, and CD11c were monitored by flow cytometry each day. The percent of cells expressing each marker is depicted vs. day of culture. The mean and standard deviations of three independent experiments are shown. B) Flow cytometric plots of co-expression of Ly6C vs. CD115 by bone marrow cells cultured in GM-CSF over seven days. C) Compiled data from three independent experiments illustrating the relative percentages of each of the four populations over 7 days of culture.

To design a strategy for isolating myeloid cells at distinct stages of GM-CSF-driven development and differentiation, we first set out to identify cell surface markers expressed with distinct kinetics during differentiation in vitro. The expression of markers such as CD11b and CD11c increased gradually and remained high through the end of the culture period, making these markers poor candidates for identifying cells at the early stages of development. However, Ly6C and CD115 were transiently expressed and with distinct kinetics. Ly6C expression peaked around day 3 and CD115 at day 5 (Fig 1A).

Two-parameter analysis of Ly6C vs. CD115 (CSF1-R) expression allowed for isolation of four distinct populations: Ly6C^-^CD115^-^, Ly6C^+^CD115^-^, Ly6C^+^CD115^+^, and Ly6C^-^CD115^+^ across the seven day culture period (Fig 1B). We then monitored the relative frequencies of each of the four populations within the culture over the same time frame (Fig 1C). The Ly6C^-^CD115^-^ population was the most common population at day 1, but decreased in frequency through day 5. Interestingly, there was an increase in this population at day 6 and 7. The Ly6C^+^CD115^-^ population was also abundant on day 1 then it decreased slowly in frequency through day 7. The Ly6C^+^CD115^+^ population was present at a low frequency initially, but became the predominant population at day 3 through 7. The Ly6C^-^CD115^+^ population was the least abundant initially, but it grew steadily through day 7 (Fig 1C).

### Myeloid cells express Ly6C and CD115 in a sequential pattern during differentiation

**Fig 2:**
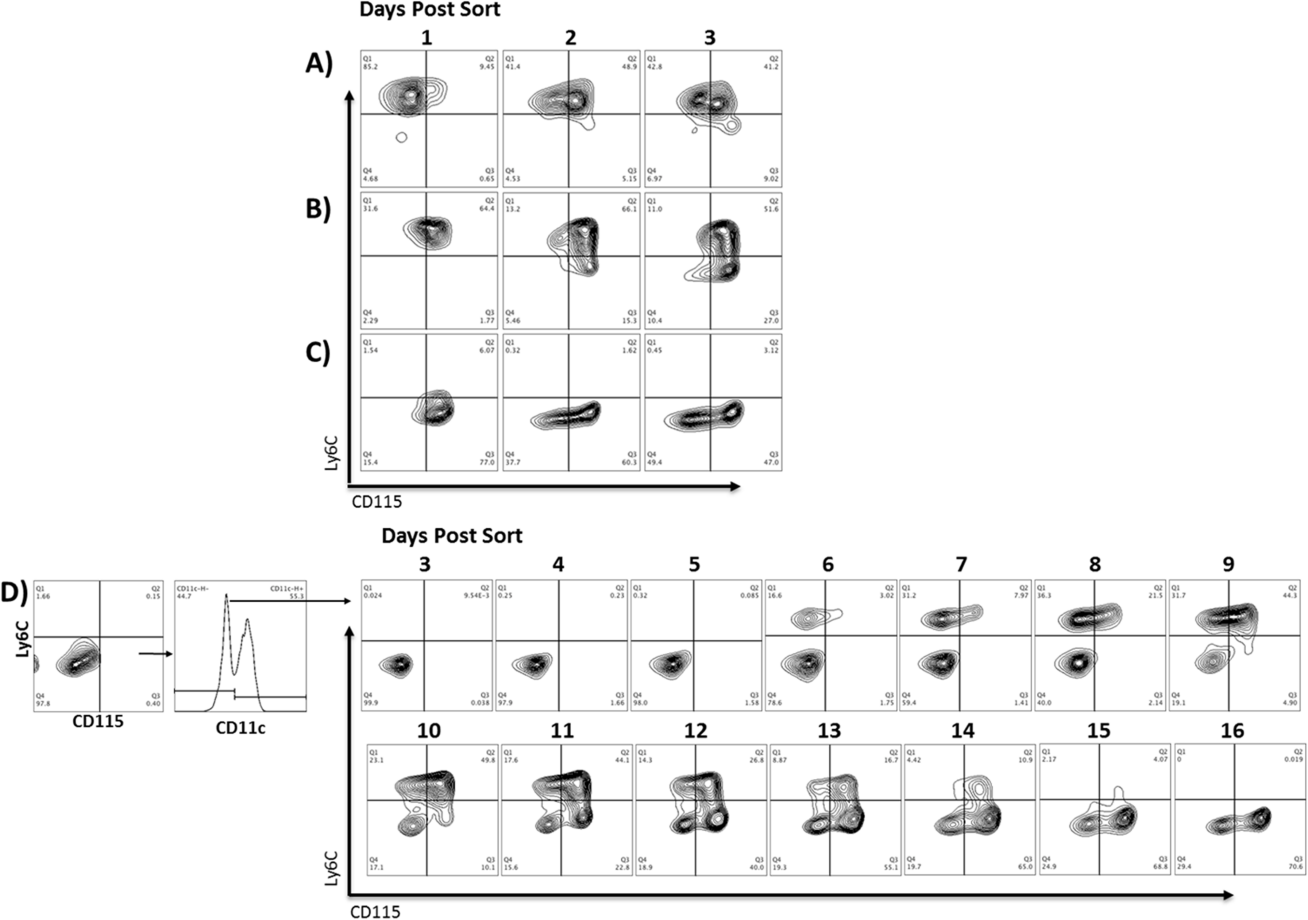
GM-CSF differentiated cells isolated based on expression of Ly6C and CD115 transition through a series of successive stages of development. After 3 days of culture in GM-CSF, cells were sorted into four populations based on expression of Ly6C and CD115. Isolated populations were then re-cultured in GM-CSF and re-examined for expression of Ly6C and CD115 on the indicated days. A) Ly6C^+^CD115^-^ B) Ly6C^+^CD115^+^ cells C) Ly6C^-^CD115^-^ cells were sorted and monitored over three days post sorting. D) Ly6C^-^CD115^-^ cells were first depleted of CD11c+ cells to remove contaminating DC. Then they were monitored over 16 days because no progression was evident within the three day time period of the other populations. Changes in the population became evident at day 7 of culture after sorting and continued through day 16.

To determine the developmental sequence of Ly6C and CD115 expression on GM-CSF-driven myeloid cell differentiation, cells were sorted on day 3 of culture into four populations based on expression of these markers. Following isolation, the purified populations were re-cultured in GM-CSF supplemented media to track their subsequent progression (Fig 2). Within one day post sorting, a subset of the Ly6C^+^CD115^-^ population up regulated expression of CD115 (Fig 2A). After two days, a majority of these cells now expressed both Ly6C and CD115 with a subset having progressed to the Ly6C^-^CD115^+^ phenotype. Following a similar pattern, cells initially expressing both markers (Ly6C^+^CD115^+^) began to down regulate Ly6C within 48 hours, and a subset of these cells went on to down regulate CD115 by 72h, transitioning to the double negative phenotype (Fig 2B). Finally, many of the Ly6C^-^CD115^+^ cells down regulated CD115 within 48 hours, with roughly half of the cells having shifted to double negative by 72h (Fig 2C).

Upon further examination of the Ly6C^-^CD115^-^ population, we identified both CD11c^+^ and CD11c^-^ cells with this phenotype. To address the GM-CSF-driven developmental potential of both populations, they were further sorted based on expression of CD11c and re-cultured (Fig 2D). The Ly6C^-^CD115^-^ CD11c^+^ population did not change its phenotype and did not proliferate in culture. In fact, within 3 days post sorting, most cells in this population were apoptotic (data not shown). The phenotype of the Ly6C^-^CD115^-^ CD11c^-^ population did not change until day 7 of culture (3 days post sort), demonstrating primarily proliferative activity during this time (data not shown). Subsequently, these cells first up regulated Ly6C, then went on to co-express CD115, and then down regulated Ly6C. At the late timepoints of 15 and 16 days post sort, many of the cells had progressed to the double negative phenotype, while a majority maintained CD115 expression. Thus, over the course of 16 days of culture, we identified at least five stages of development, ending with two distinct CD11c+ populations (Ly6C^-^CD115^-^ and Ly6C^-^CD115^+^) (Fig 2D).

To control for any potential off-target effects of high-speed cell sorting on the cell populations, we utilized a magnetic separation method (MACS, Miltenyi) as an alternative approach. Using a negative selection method, we acquired Ly6C^-^ CD115^-^ cells at 92–96% purity. After separation, these cells were cultured with GM-CSF for 12 days to follow their progression (S2 Fig). Consistent with the results observed in Fig 2, the cells first up-regulated Ly6C, then CD115, then down regulated Ly6C, and then down regulated CD115 (S2 Fig). Having observed the same pattern of development in the absence of cell sorting, we conclude that sorting did not significantly alter the developmental progression of the cells.

### Developmental progression of GM-CSF driven differentiation in vitro and in vivo

**Fig 3:**
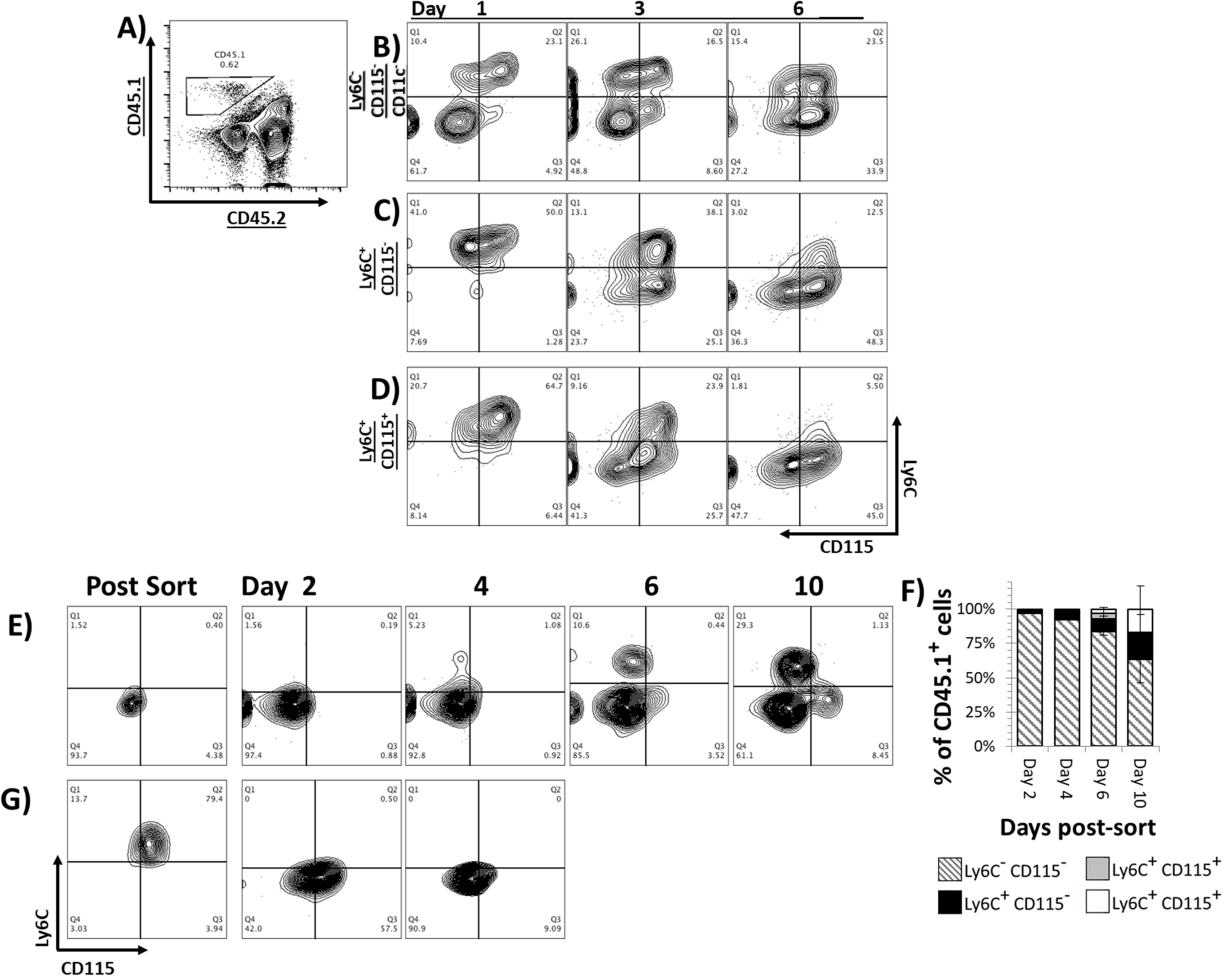
Developmental progression of GM-CSF driven differentiation in the presence of feeder cells in vitro or in vivo. Bone marrow was harvested from Ptprc^b^ (CD45.1) mice, cultured in GM-CSF supplemented media for 2 or 5 days, and sorted as previously described. **A)** 10^4^ CD45.1^+^ sorted cells were co-cultured with 10^6^ CD45.2^+^ fresh bone cells, and **B-D)** Ly6C/CD115 expression was analyzed for six days by flow cytometry. Adoptive transfers were performed by intraperitoneal injection of 10^6^ (CD45.1^+^) **E)** Ly6C^-^CD115^-^ or **G)** Ly6C^+^CD115^+^cells into CD45.2 mice, suspended in PBS with 200ng of GM-CSF. **F)** Composition of recovered CD45.1^+^ cells following CMP adoptive transfer 2, 4, 6, and 10 days post injection compiled from 3 independent experiments. Mice received daily injections of 200ng of GM-CSF. Peritoneal lavage was collected every 48 hours, and donor (CD45.1^+^) cells Ly6C/CD115 levels were evaluated by flow cytometry.

To first determine if the same sequence of phenotypic development was observed in the presence of other bone marrow cells, we isolated 3 of the earlier populations by sorting at day 3 of culture. These CD45.1-expressing cells were then mixed in culture with an excess of congenic (CD45.2) bone marrow cells and GM-CSF for up to 6 days. The expression of Ly6C and CD115 on the CD45.1-expressing cells was measured at days 1, 3, and 6 post sorting (Fig 3A–3D). In a pattern similar to that observed in Fig 2, double negative cells first up-regulated Ly6C, then CD115, then the majority of the cells became Ly6C^-^CD115^+^ by day 6 (Fig 3B). Likewise, Ly6C^+^CD115^-^ cells first up-regulated CD115, then the majority of cells displayed a CD115 single positive phenotype by day 6 (Fig 3C). Ly6C^+^CD115^+^ cells quickly down-regulated Ly6C, and the majority of cells became double negative by day 6 (Fig 3D). The one most notable difference between this and our previous experiment was the kinetics of development. In the presence of the other bone marrow cell populations, the developmental sequence proceeded in the same order, but did so much more rapidly (Figs 2 and 3).

To determine if a similar GM-CSF-driven developmental sequence of myeloid differentiation was observed in vivo, we used an adoptive transfer system in which congenic donor cells could be tracked in recipient mice. Bone marrow was isolated from mice expressing the CD45.1 isoform and expanded in culture with GM-CSF for 2 or 5 days (to increase the yield of cells at early vs. later stages of development). These cells were then sorted based on expression of Ly6C and CD115 and transferred into mice expressing the CD45.2 isoform. GM-CSF was administered daily for the indicated time points. Cells were then harvested from the peritoneal cavity and spleen. Expression of Ly6C and CD115 was then measured on CD45.1-expressing cells (Fig 3E–3G). The Ly6C^-^CD115^-^ cells began to upregulate expression of Ly6C after 4 days in vivo and this population increased at day 6 and day 10 (Fig 3E). These double negative cells also gave rise to cells with a Ly6C^-^CD115^+^ phenotype, first appearing at day 6, and increasing at day 10 post transfer. The pattern of expression that was consistently observed was first Ly6C, followed by CD115 in each case, giving rise to a similar progression as observed in vitro (Fig 3E). To our surprise however, we were unable to recover many cells with the monocyte phenotype (Ly6C^+^CD115^+^) in these experiments. This could have been due to their highly migratory function or rapid transition to the next stage of development in vivo. To investigate the fate of monocytes in vivo, we transferred in Ly6C^+^CD115^+^ cells and looked for them at 2 and 4 days post transfer (Fig 3G). Again, we were not able to recover cells with the Ly6C^+^CD115^+^ phenotype even at day 2, indicating that they had either migrated out of the site or had all transitioned to the next stage of development.

### Phenotypic characterization of each population based on cell surface markers and gene expression

**Fig 4:**
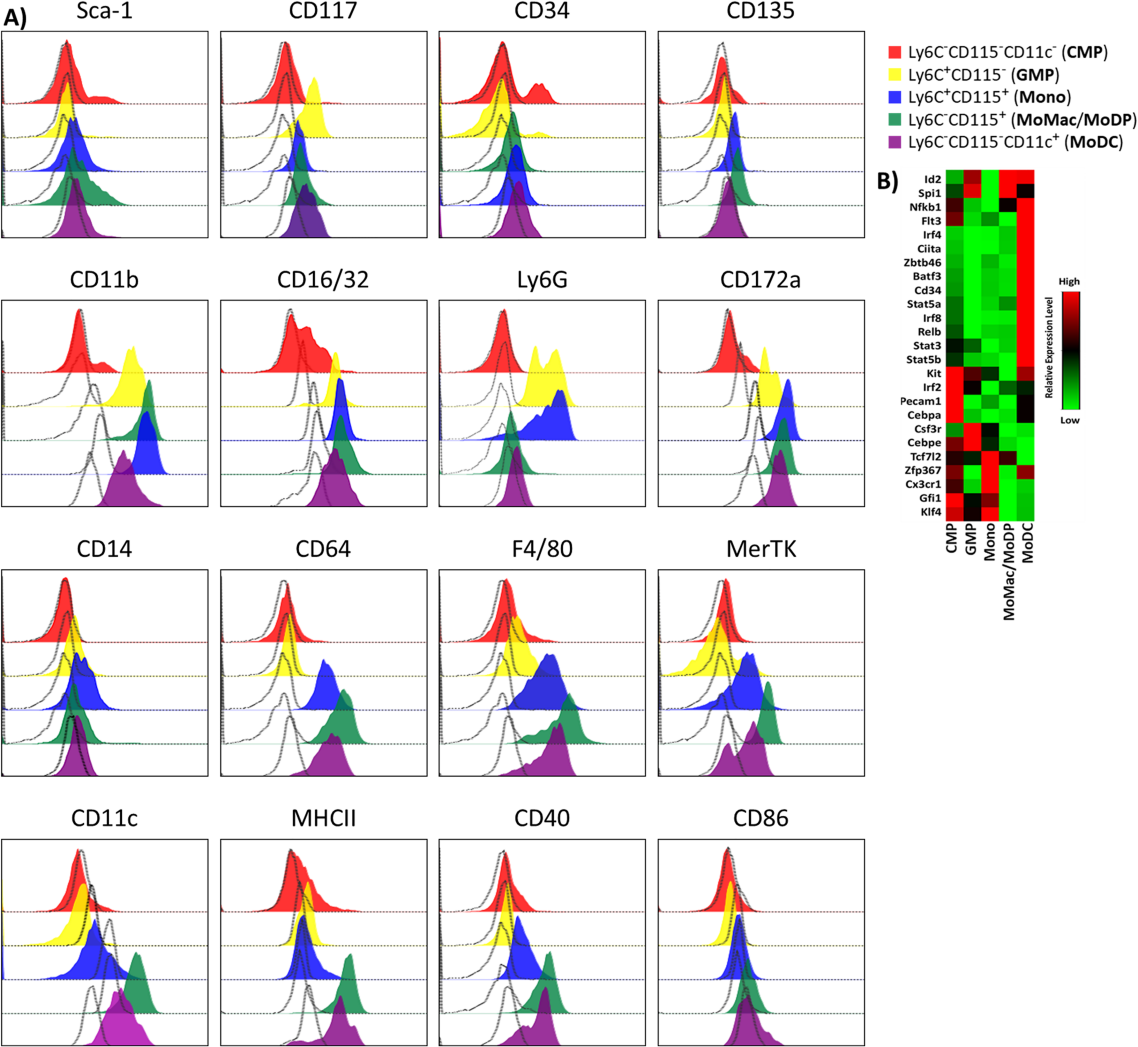
Distinct cell surface marker and gene expression profiles in the five stages of inflammatory DC development. **A)** 16 cell surface markers were measured by flow cytometry at each of the five stages of development. Empty histograms represent fluorescence minus-one controls. **B)** After three days of culture in GM-CSF, bone marrow cells were sorted into five populations based on expression of Ly6C, CD115 and CD11c. RNA was purified from each population and analyzed for expression of 25 genes plus controls using a custom qRT-PCR array. Relative levels of expression are depicted by intensity of color on the heat map with red being highest expression and green lowest. Results represent averages from three independent experiments.

Each of the five populations were phenotypically characterized based on expression of cell surface markers representing several stages of myeloid cell development [31–33] (Fig 4A) and on patterns of expression of key genes active at different stages of development (Fig 4B). To identify the earliest progenitor populations, we monitored expression of Sca-1, CD117, and CD34. Flt3L-responsive DC progenitors were tagged with anti-CD135. CD11b was measured as a marker of myeloid commitment, CD16/32 was measured to differentiate CMP (which lack its expression) from GMP and all subsequent populations (which express it). Ly6G was tested to identify granulocytes and their precursors [34]. CD14, CD64, F4/80, and MerTK were tested as markers of macrophages. Markers of later stage DC development included CD172a (SIRP-α) [35], CD11c, CD86 and MHC class II (Fig 4A). We then measured the expression of 24 targeted genes using a custom qRT-PCR array (Fig 4B). We selected genes known to be expressed by cells at several stages of myeloid development [31–33]: CMPs (Gfi1, Kit, Cebpa, Flt3, Cd34), GMPs (Csf3r, Cebpe, Spi1), monocytes (Tcf7l2, Cx3cr1, Pecam, Klf4), and during conventional DC development (Irf2, Id2, Irf8, Stat3, Stat5, Nfkb, Relb, Batf3, Irf4, Ciita). Data are depicted as a heat map showing relative expression levels (Fig 4B).

Ly6C^-^CD115^-^CD11c^-^ (CMP): This early population contained a subset of very early progenitors expressing Sca-1, and CD34, with generally lower levels of expression of all of the subsequent markers (Fig 4A and S3 Fig). These cells were unique in lacking CD11b and expressed low levels of CD16/32, which were expressed by all subsequent populations. They also expressed little or none of the macrophage and DC markers (Fig 4A and S3 Fig). The gene expression profile of these cells demonstrates expression of genes typical of early myeloid progenitors, monocyte committed cells and Common Myeloid Progenitors: Gfi1 [36], Klf4 [37], Cebpa [38,39], Pecam1 [40], Irf2 [41] and Kit [26]. Taken together, the cell surface profile and gene expression pattern indicates that these Ly6C^-^CD115^-^CD11c^-^ cells most closely correspond to Common Myeloid Progenitors (CMP).

Ly6C^+^CD115^-^ (GMP): This population lacked expression of the most of the stem cell markers, except for a small subset that expressed CD34 (Fig 4A and S3 Fig). This population was also the first to demonstrate higher levels of expression of CD11b, CD16/32, Ly6G, and CD172a than CMP. These cells lacked expression of the macrophage markers CD14, CD64, low F4/80, and low MerTK. These cells also expressed very low levels of the dendritic cell markers, CD11c, MHC class II, CD40 and CD86 (Fig 4A and S3 Fig). Gene expression analysis revealed expression of Csf3r and Cebpe [42], which are hallmarks of Granulocyte Macrophage Progenitors (GMPs), as well as Spi1 (PU.1) [43] (Fig 4B). Thus, this Ly6C^+^CD115^-^ population closely resembles GMPs.

Ly6C^+^CD115^+^ (Monocytes): These cells lacked expression of the stem cell markers Sca-1, CD117, and CD34, and expressed low levels of CD135. The majority of cells in this population also expressed Ly6G cells yet at a lower frequency than GMP (Fig 4A and S3 Fig). This population expressed a very low level of CD11c and a high level of CD172a. This population displayed intermediate CD40 and CD86, yet low level MHC class II. Notably, the Ly6C^+^CD115^+^ cells were the first population to demonstrate high expression of the macrophage markers, CD64 and F4/80, yet intermediate expression of MerTK. These cells also expressed high levels of monocyte-associated genes Tcf7l2 [44], Klf4, and Cx3cr1 [45] (Fig 4B). Collectively, the phenotype and gene expression pattern most closely resemble the monocyte cell type.

Ly6C^-^CD115^+^ (moMac): Cells with this phenotype were negative for stem cell markers and Ly6G expression. Notably, this population expressed the highest levels of macrophage markers F4/80, and MerTK relative to the other four populations. They also expressed high levels of CD11c, MHC II, and CD40 and an intermediate level of CD86 (Fig 4A and S3 Fig). Upon examination of gene expression, these cells displayed high level expression of only two genes, Spi1 and ID2 [31]. Spi1 (PU.1) is a central transcription factor in myeloid cell and DC development [46,47]. While highly expressed in the Ly6C^-^CD115^+^ population, Spi1 was also up regulated initially in the Ly6C^+^CD115^-^ population (Fig 4B). Based on the phenotype and gene expression patterns, this population most closely resembles monocyte-derived Macrophages (moMac). A similar population, referred to as GM-Macrophages, was recently described by Helft, et al. [30]

Ly6C^-^CD115^-^CD11c^+^ (moDC): This final population expressed CD11c and CD172a as well as high levels of MHC class II, CD40, and CD86. However, these cells displayed low levels of the macrophage markers CD14, CD64, F4/80, and MerTK and had a slightly lower level of CD11b expression (Fig 4A and S3 Fig). This population also expressed high levels of several genes critical for DC function and differentiation including: Ciita (required for transcription of MHC class II genes); Stat5a, Stat5b, and Stat3 (transcriptional regulators of myeloid differentiation and GM-CSF signaling [31]); Zbtb46 and Batf3, (both critical to DC development [48,49]); and Relb and Nfkb1, both well documented regulators of inflammatory gene expression (Fig 4B). Also, while we were unable to detect Flt3 expression by flow cytometry on this population, there was a strong signal of its gene expression. Taken together, this population closely reflects monocyte-derived Dendritic Cells (moDC) in phenotype.

### The moMac population contains DC-precursors as well as macrophages

**Fig 5:**
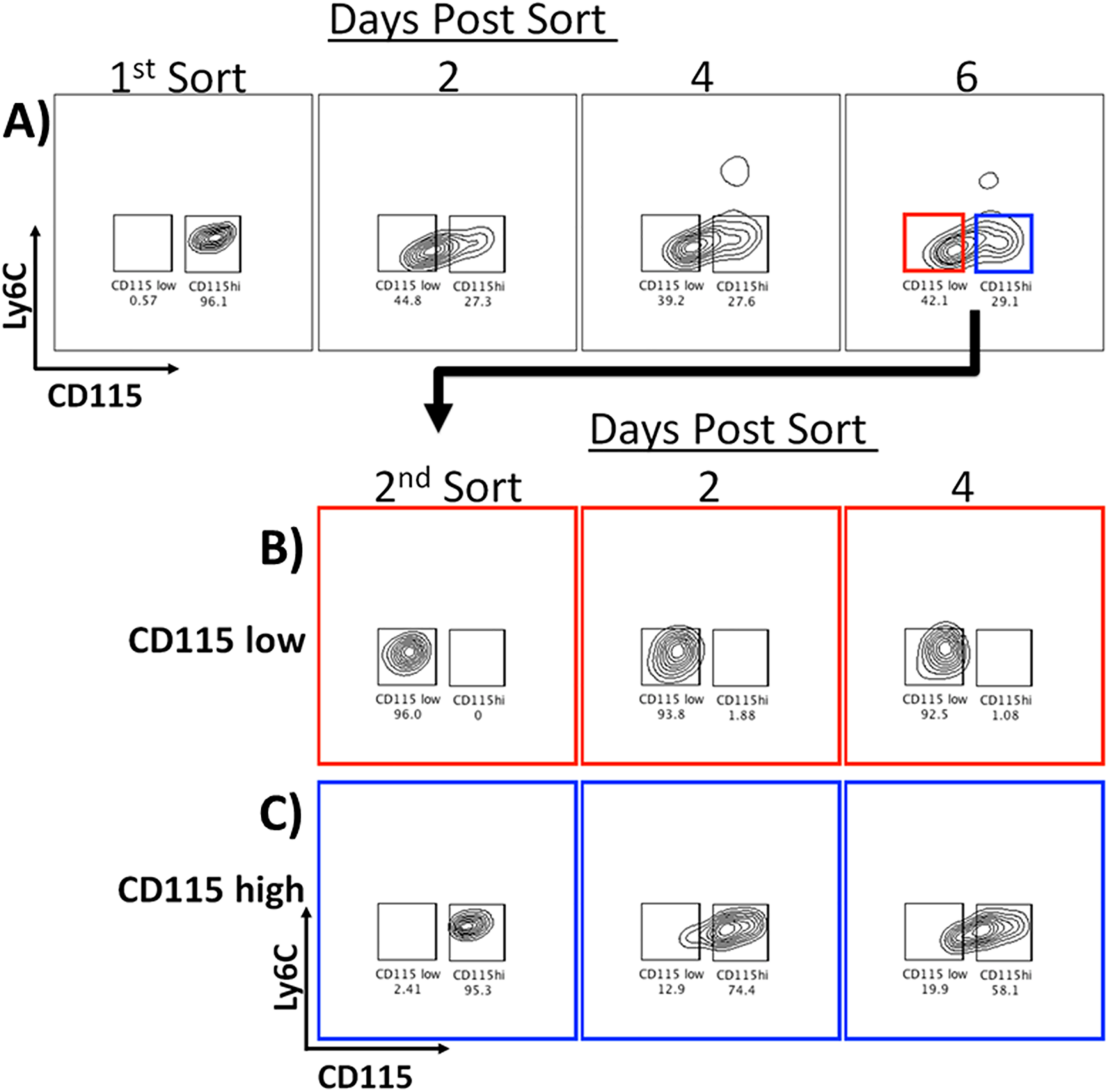
Early moMacs give rise to two cell types. **A)** After five days of culture in GM-CSF, moMacs (Ly6C^-^CD115^+^) were purified and recultured with GM-CSF. CD115 expression was monitored by flow cytometry for 6 days. Six days post-initial sort, **B)** moDC (Ly6C^-^CD115^-^) and **C)** moMacs (Ly6C^-^CD115^+^) were purified and recultured with GM-CSF. CD115 expression was monitored by flow cytometry for the next 48 hours.

We routinely observed that most Ly6C^-^CD115^+^ cells would ultimately down regulate CD115, taking on the phenotype of monocyte-derived DC (moDC). However, in long-term culture, a subset of Ly6C^-^CD115^+^ (moMac) persisted, maintaining CD115 expression even out to 16 days (Figs 2 and 3). To more definitively address these final stages of development, we performed a two-stage sorting experiment. moMacs were first purified from GM-CSF stimulated bone marrow on day 5, and re-cultured for 6 days in GM-CSF (Fig 5A) before undergoing a second sort, based on their level of CD115 expression (Fig 5B and 5C). 48 hours after the first sort, ~44% of moMacs had downregulated CD115, and this phenotypic distribution did not change over the subsequent 4 days (Fig 5A). Six days after the first sort, cells that were selected based on low level of CD115 expression maintained that phenotype (Fig 5B). Likewise, the majority of CD115^high^ cells maintained CD115 expression over this time period (Fig 5C) yet, a small subset within this population continued to give rise to CD115 low cells (~20% by 4 days).

**Fig 6:**
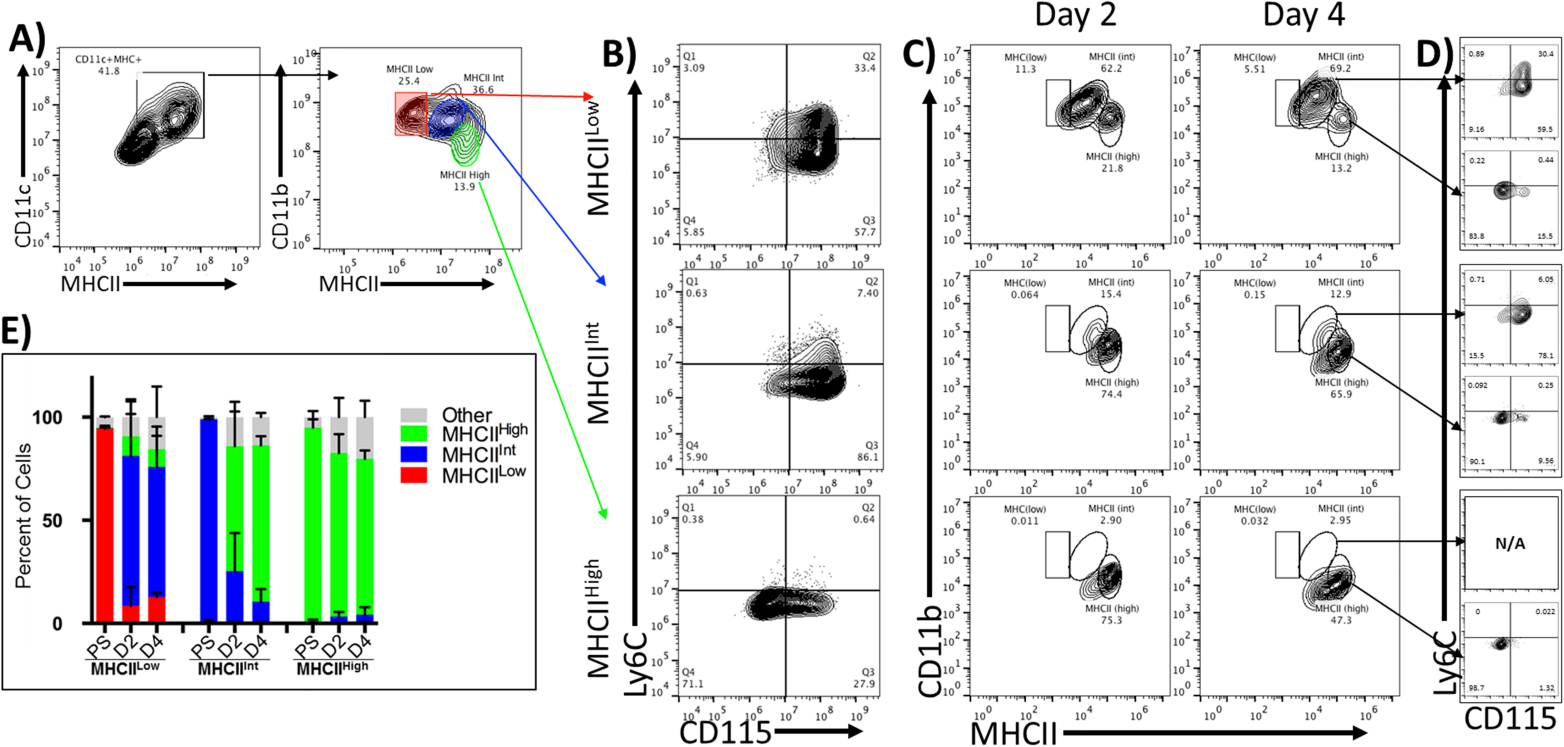
MHC class II level distinguishes developmental stages within moMac phenotype. Bone marrow cells were cultured in GM-CSF for 5 days. **A)** CD11c^+^ cells were sorted based on expression of CD11b and MHCII into three populations: MHCII^Low^, MHCII^Int^, and MHCII^High^. **B)** Expression of Ly6C and CD115 were analyzed post sort, and the isolated populations were re-cultured in GM-CSF. **C)** Changes in CD11b and MCHII expression were analyzed on day 2 and 4 post sort by flow cytometry. **D)** Resulting MHCII^Int^ and MHCII^High^ cells on Day 4 were further analyzed by Ly6C and CD115 expression by flow cytometry. **E)** Percent of cells exhibiting MHCII^Low^, MHCII^Int^, or MHCII^High^ phenotypes post-sort (PS) and after re-culture for 2 or 4 days.

To determine if the moDC progenitors could be further distinguished from moMacs based on CD11c, CD11b, and MHCII expression, we incorporated a sorting strategy previously published by Helft et al [30]. After five days in culture, 41% of cells were CD11c^+^. Within this group, there were three CD11b^+^ populations: MHCII^low^ (29%), MHCII^int^ (34.3%), and MHCII^high^ (17.2%) (Fig 6A).

Post-sort analysis of the MHCII^low^ population revealed two predominant populations, Ly6C^+^CD115^+^ and Ly6C^-^CD115^+^ (Fig 6B; top panel). Two days after sorting, most MHCII^low^ cells had upregulated MHCII to an intermediate level (72.6% ± 27.4 on day 2; 62.2% ± 19.6 on day 4) (Fig 6C; top panel). Additionally, analysis of these cells’ Ly6C and CD115 levels four days post sort showed the resulting MHCII^int^ cells represent a heterogenous mixture of primarily Ly6C^+^CD115^+^ (33.6% ± 4.6) and Ly6C^-^CD115^+^ (52.8% ± 9.47) (Fig 6D). A small subset of MHCII^low^ cells were also able to give rise to MHCII^high^ cells (9.5% ± 10.7 on day 2; 8.58% ± 6.5 on day 4) (Fig 6C; top panel). Unlike the MHCII^int^, MHCII^high^ were primarily (80.4%±4.81) Ly6C^-^CD115^-^ (Fig 6D; top panel). The results of several replicate experiments are graphically illustrated in Fig 6E.

The isolated MHCII^int^ cells were primarily of the Ly6C^-^CD115^+^ phenotype (86.1%), with few exhibiting the Ly6C^+^CD115^+^ phenotype (7.4%) (Fig 6B; middle panel). After 48 hours of culture in GM-CSF, nearly all MHCII^int^ cells had upregulated MHCII to a high level (60.6% ± 21.5 on day 2; 75.75% ± 4.45 on day 4), while only a fraction maintains the MHCII^int^ phenotype (14% ± 2.7 on day 2; 13.69% ± 2 on day 4) (Fig 6C; middle panel). The newly developed MHCII^high^ cells primarily consisted of Ly6C^-^CD115^-^ cells (91.8% ± 2.47), whereas the 72.75% ± 7.57 of those that maintained MHCII^int^ phenotype exhibited a Ly6C^-^CD115^+^ phenotype (Fig 6D; middle panel).

Finally, analysis of MHCII^high^ isolated cells showed that they maintained their MHCII^high^ phenotype on day 2 and day 4 (Fig 6C; bottom panel). Additionally, 97.9% ± 0.14 of these cells exhibited a Ly6C^-^CD115^-^ phenotype 4 days post isolation. Together, these patterns suggest that, when isolated on day 5, CD11c^+^CD11b^+^MHCII^low^ cells act as a progenitor to a terminal Ly6C^-^CD115^+^MHCII^int^ population, resembling moMacs. CD11c^+^CD11b^+^MHCII^int^ primarily give rise to Ly6C^-^CD115^-^MHCII^high^ cells, suggesting these cells have moDC precursor activity and thus we refer to them as monocyte-derived Dendritic Precursor (moDP). Finally, cells with the phenotype CD11c^+^CD11b^+^MHCII^high^ tended to maintain high MHCII expression, indicative of a DC phenotype.

## Discussion

**Fig 7:**
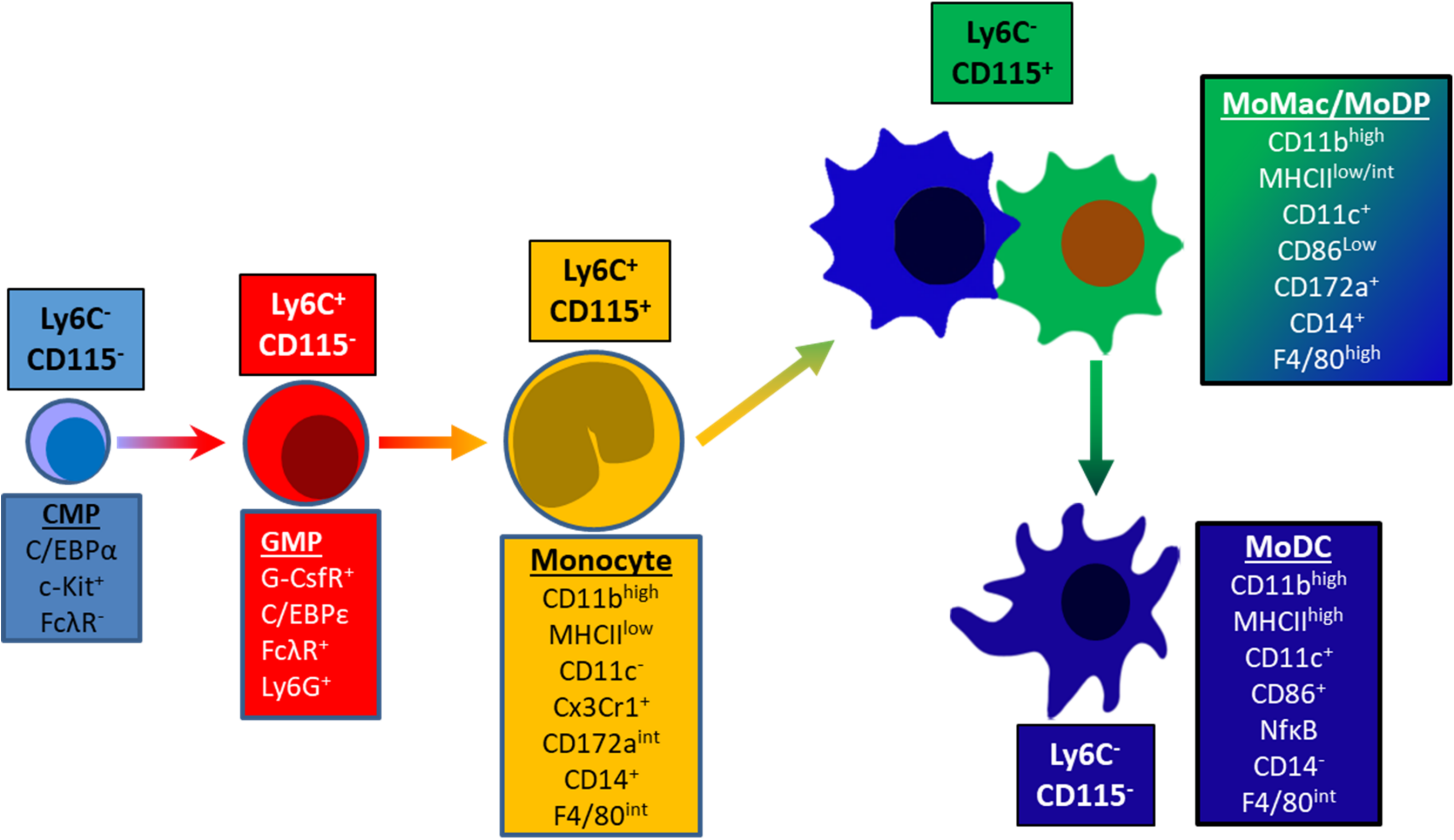
Comprehensive model of GM-CSF driven DC development. Transcriptional and phenotypic changes as cells progress through GM-CSF driven development. Common myeloid progenitor gives rise to granulocyte/monocyte progenitor (GMP), followed by monocytes, and monocyte-derived macrophages (moMac). moMacs are maintained long term and share a phenotype with a precursor of monocyte-derived DC (moDC). This precursor has been termed monocyte-derived DC progenitor (moDP).

Based on these findings, we propose that GM-CSF-driven differentiation of murine bone marrow cells in vitro proceeds through at least five distinct stages: Common Myeloid Progenitor (CMP), Granulocyte/Macrophage Progenitor (GMP), Monocytes, monocyte-Derived Macrophage/monocyte-derived Dendritic Precursor (moMac/moDP) and monocyte-derived DC (moDC) (Fig 7). Three of the stages of development are “transitional” indicating that by day 6 of culture they are absent or represent only a very small subset of the cells (CMP, GMP, Monocytes). The two dominant populations by day 6 represent differentiated cell types, the phenotypes of which are maintained long term (moMac and moDC). The moMac population was also found to contain a population of moDC precursors (moDP) that shares most phenotypic features with moMac. These cells were distinguishable only by their intermediate level of MHC class II on day 5 and their developmental plasticity (Fig 6). These data further demonstrate that these isolated populations have distinct expression profiles of key genes and phenotypic markers involved in myeloid and DC development, supporting the notion that they represent distinct stages of the developmental process driven by GM-CSF.

The first population, Ly6C^-^CD115^-^CD11c^-^ cells correspond to a common myeloid progenitor (CMP) population based on their ability to give rise to all subsequent populations both in vitro and in vivo (Fig 2 and Fig 3), and their expression of early progenitor markers (Sca-1, c-kit, and CD34) and lack of FcγR [26]. The Ly6C^+^CD115^-^ population appears second in the developmental progression and displays phenotype typical of granulocyte monocyte/macrophage progenitor cells (GMP) based on their expression of CSF3R, CEBPe, Ly6G, and FcγR (Fig 4) [50].

The third population in the GM-CSF-driven developmental progression is representative of monocytes, expressing both Ly6C and CD115, as well as CX_3_CR1. These cells were also the first to demonstrate expression of the macrophage markers, CD64 and F4/80, yet lacked MerTK expression. In contrast, these cells did not express a key marker of DC maturation and function, MHC class II. The gene expression pattern of monocytes was also quite distinct from both GMPs and moMac. Others have shown that during *Listeria monocytogenes* infection and other conditions in which GM-CSF is at high levels in circulation, Ly6C^high^ monocytes differentiate into TNF/iNOS producing DCs (Tip-DC) [11,51,52]. However, new evidence suggests that Tip-DC respond more specifically to M-CSF than GM-CSF [53]. Nonetheless, we found that after TLR triggering, Ly6C^+^ CD115^+^ cells were able to produce TNF-α and iNOS (unpublished data).

A recent study by Hettinger, et al identified a common monocyte progenitor (cMOP) in mouse bone marrow that gives rise to monocytes and macrophages [19]. This population proliferated in response to GM-CSF as well as IL-3, and M-CSF [19]. While some of the phenotypic features of these cells were shared with specific populations identified in our cultures, there were several inconsistencies. As a progenitor cell type, cMOP express CD117 (c-kit) and lack CD11b, an expression pattern exclusive to CMP in our system. However, cMOP also express CD115, Ly6C, and CX_3_CR1, much like the monocytes identified in our system. Thus, cMOP do not directly correspond to any population identified in our cultures. One likely explanation for why these cells were not detected in our system, is that cMOP were isolated from whole bone marrow while our cells were treated in culture with GM-CSF for several days, likely selecting for non-progenitor populations. cMOP are also a very rare population, and could have been overlooked by our sorting strategy.

Following adoptive transfer of CMP in vivo, we sequentially recovered cells phenotypically resembling all of the developmental stages observed in vitro, with the exception of monocytes. We did however, recover a large number of moMacs by day 10 post transfer, suggesting that these cells might have rapidly transitioned through the monocyte stage, yet were not detected at the timepoints we tested. An alternative explanation is that cells at the monocyte stage of development migrated out of the peritoneal cavity. Thus, we examined the spleen, blood, and bone marrow for the presence of these cells, yet were not able to detect them (data not shown). In support of the notion that monocytes rapidly transition into DC, our in vivo data demonstrate that when cells at the monocyte stage were transferred, they rapidly underwent transformation into moMac and moDC in the recipient, such that virtually no monocytes are detectable after 48h (Fig 3). These findings are in line with other studies demonstrating the rapid differentiation (~18h) in vivo into DC or macrophages based on available space in the niche. Thus, the kinetics of monocyte differentiation in vivo appear to be more rapid than in vitro [54,55].

The fourth population, Ly6C^-^CD115^+^ (moMac), contains two populations; one precursor that gives rise to DC, and one macrophage-like cell that is maintained long term (Fig 6). These results are generally consistent with a recent report by Helft, et al, with some minor differences [30]. They demonstrated that after six days culture in GM-CSF there were two CD11c^+^MHCII^+^ populations: one CD115^+^ GM-MACs (similar to moMac), and a second CD135^+^ GM-DC. These cells could also be distinguished based on level of MHC class II and CD11b. When we utilized CD11b and MHC II sorting strategy, we observed 3, not 2, populations: those with low, intermediate, or high level MHCII expression. The MHC^low^ cells reliably corresponded to moMac, retaining an intermediate MHC II expression. The MHC^int^ population demonstrated strong precursor activity, corresponding to moDP. Finally, the MHC^hi^ population maintained their phenotype, corresponding to moDC. While the level of expression of MHC class II serves as a strong predictive factor of cell type, determining the full extent of the differences in these three populations is the focus of ongoing studies. Notably, moMacs are also distinct from Ly6C^low^ monocytes described in the literature in that they have intermediate basal levels of CD86 and have a larger morphology than monocytes (data not shown) [56].

Common dendritic cell precursors (CDPs) and pre-DCs have been identified as giving rise to conventional and plasmacytoid DCs respectively during development. We believe we have now identified a cell type driven by GM-CSF that shares many of phenotypic features of moMacs, yet acts as a precursor of moDC. moDP also share several phenotypic features with CDPs: both express CD172a^int^CD115^+^, but moDP are CD11c^+^, more similar to pre-DCs. There has been little functional analysis of CDPs, but, unlike moDP, they have been observed to have low MHCII [24]. Unlike CDP and pre-DCs, moDP also express high levels of CD11b. Additionally, it has been previously shown that CDPs do not originate from monocytes [25,57]. Another recent study has demonstrated the presence of one or more monocyte derived DC precursors in the skin (P2MoDC) [58]. However, while both cell types are similar in developmental status, moDP are distinct different from P2MoDCs in regard to Ly6C, MHCII, and CD11c expression. There are likely several tissue-specific factors that dictate different phenotypes and functions in vivo that would not be observed in this in vitro system.

As expected, the hallmarks of DC differentiation (Fig 4) were most highly expressed in population 5, the moDC. Only the moDC expressed transcription factors typical of DC: Zbtb46, Irf4, Irf8, Batf3, as well as other DC-associated molecules such as Flt3, Ciita, Stat5a, and Stat5b. Expression of CD135 (Flt3) has been emphasized as indicative of dendritic cell phenotype or ontogeny [30]. Interestingly, we observed Flt3 expression at the transcript level, but not at the cell surface. Perhaps since GM-CSF is the sole driving cytokine in this system, Flt3 expression is accessory to moDC development.

Collectively, these findings offer several novel insights as to the diversity of cell types present in GM-CSF-driven bone marrow cultures and the timing of their progression through the developmental program to become DC. Thanks to our sorting strategy, large numbers of cells can be isolated at each of these stages for further functional analysis. This represents a step forward not only in the study of murine DC differentiation, but likely can be adapted for therapeutic applications of human DC.

## Supporting information

S1 Fig General gating strategy for the 5 cell types. Murine bone marrow stained with Ly6C and CD115 was analyzed by flow cytometry over a range of time points. Debris-size and high SSC events were excluded. Early cell types (Ly6C^-^ CD115^-^, Ly6C^+^ CD115^-^, and Ly6C^+^ CD115^+^) were collected and analyzed at early times points when they were most abundant (Day 1 and 2), where as more developed cells types (Ly6C^+^ CD115^+^, Ly6C^-^ CD115^+^, Ly6C^-^ CD115^-^) were collected and analyzed at later times points (Day 3–5). A doublet gate was applied during sorting to exclude cells that clump while waiting to be sorted. However, this was not necessary for much of our analysis, as cells were analyzed immediately after filtering.
(TIF)

S2 Fig Developmental progression of CMP purified by magnetic cell isolation. Murine bone marrow was harvest and cultured as previous described. Ly6C^-^ CD115^-^ cells were isolated on Day 2 post harvest by MACS according to manufacture’s protocol. Briefly, 3x10^6^ cells were stained with CD115-biotin and Ly6C-APC, followed by an incubation with anti-APC magnetic beads. Tagged cells were passed through a MS column. The flow through faction was incubated with anti-Biotin magnetic beads and passed through fresh MS column. The flow through contained an enriched Ly6C^-^ CD115^-^ population. These cells were analyzed for purity and re-cultured in GM-CSF supplemented media.
(TIF)

S3 Fig Mean Fluorescence Intensity (MFI) of markers commonly expressed by myeloid cells. Black bars indicated MFI of indicated cell surface markers. These are overlaid with gray bars that represent the MFI of the Fluorescence Minus One control. Populations are indicated by 1 (CMP), 2 (GMP), 3 (monocytes), 4 (moMac/MoDP), and 5 (MoDC).
(TIF)
