# Supplementary figures and images for: Analysis of the developmental stages, kinetics, and phenotypes exhibited by myeloid cells driven by GM-CSF in vitro
Source: PLoS One. 2017 Jul 27;12(7):e0181985. doi: 10.1371/journal.pone.0181985 (PMC5531556; doi:10.1371/journal.pone.0181985)

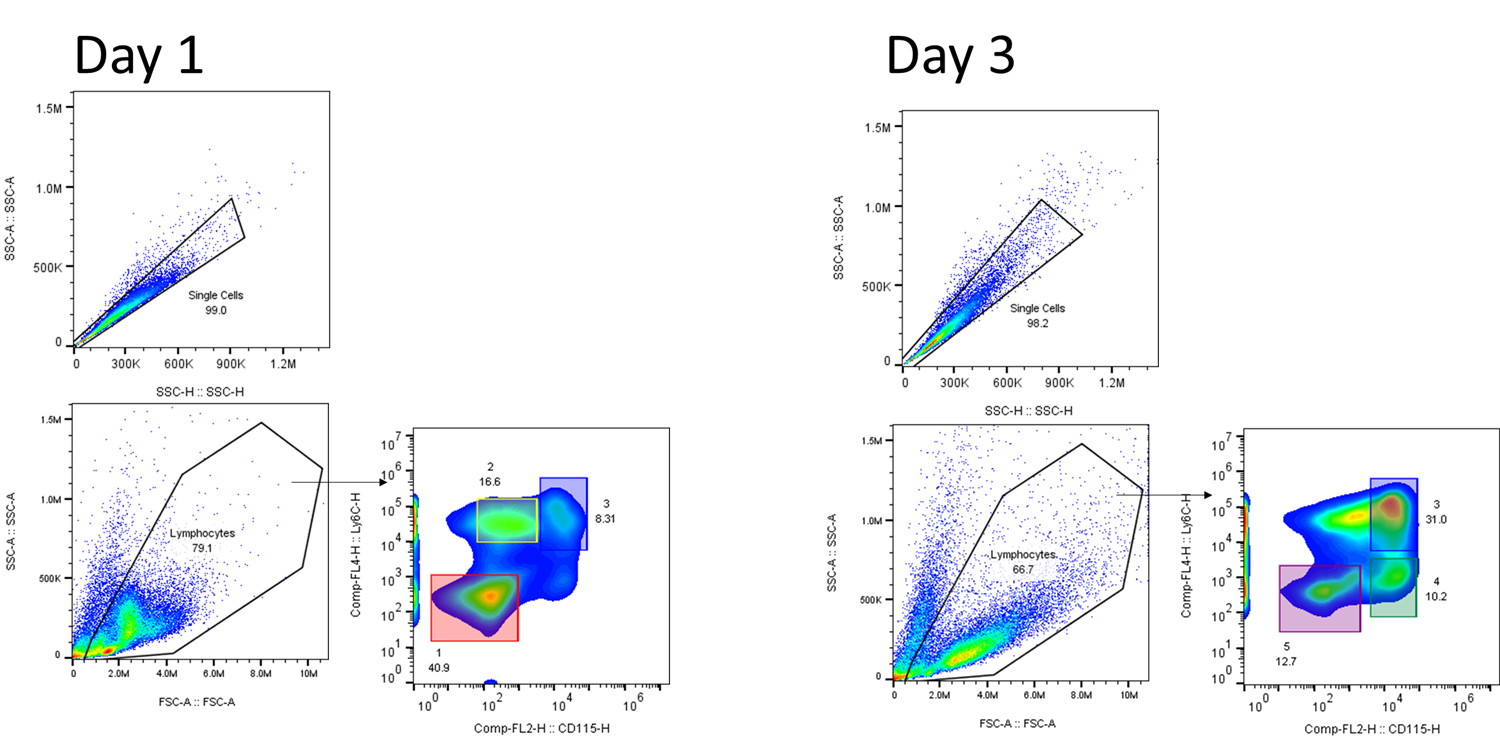

Supplement: S1 Fig — Murine bone marrow stained with Ly6C and CD115 was analyzed by flow cytometry over a range of time points. Debris-size and high SSC events were excluded. Early cell types (Ly6C- CD115-, Ly6C+ CD115-, and Ly6C+ CD115+) were collected and analyzed at early times points when they were most abundant (Day 1 and 2), where as more developed cells types (Ly6C+ CD115+, Ly6C- CD115+, Ly6C- CD115-) were collected and analyzed at later times points (Day 3–5). A doublet gate was applied during sorting to exclude cells that clump while waiting to be sorted. However, this was not necessary for much of our analysis, as cells were analyzed immediately after filtering. (TIF) [file pone.0181985.s001.tif]

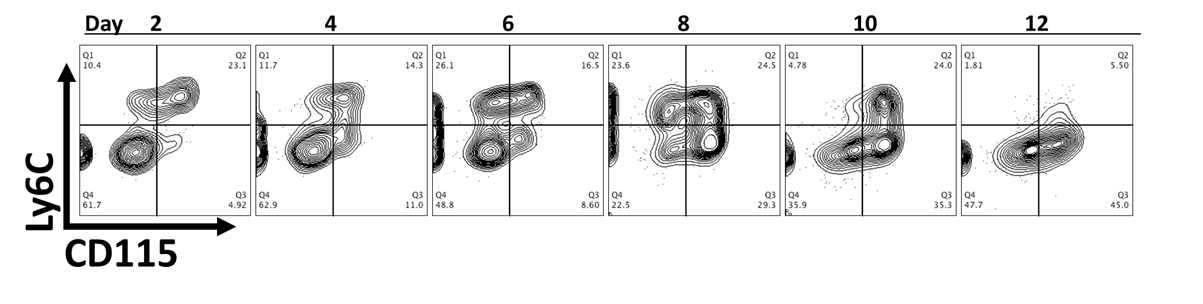

Supplement: S2 Fig — Murine bone marrow was harvest and cultured as previous described. Ly6C- CD115- cells were isolated on Day 2 post harvest by MACS according to manufacture’s protocol. Briefly, 3x10^6^ cells were stained with CD115-biotin and Ly6C-APC, followed by an incubation with anti-APC magnetic beads. Tagged cells were passed through a MS column. The flow through faction was incubated with anti-Biotin magnetic beads and passed through fresh MS column. The flow through contained an enriched Ly6C- CD115- population. These cells were analyzed for purity and re-cultured in GM-CSF supplemented media. (TIF) [file pone.0181985.s002.tif]

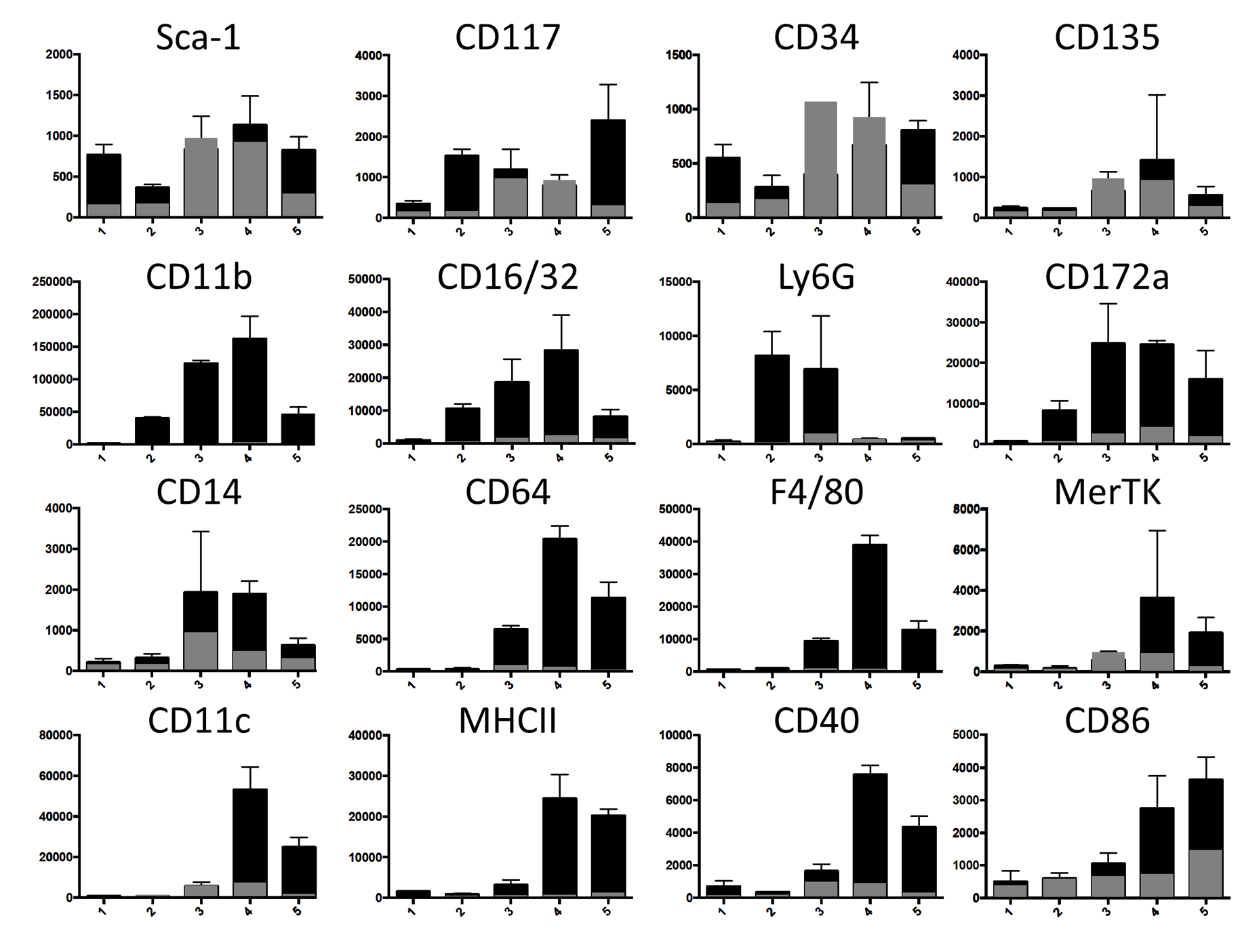

Supplement: S3 Fig — Black bars indicated MFI of indicated cell surface markers. These are overlaid with gray bars that represent the MFI of the Fluorescence Minus One control. Populations are indicated by 1 (CMP), 2 (GMP), 3 (monocytes), 4 (moMac/MoDP), and 5 (MoDC). (TIF) [file pone.0181985.s003.tif]
